# A digital patient-reported outcome (electronic patient-reported outcome) system for patients with severe psychiatric disorders: User-centered development study and study protocol of a multicenter-controlled trial

**DOI:** 10.1177/20552076231191009

**Published:** 2023-10-25

**Authors:** Caspar Wiegmann, Esther Quinlivan, Twyla Michnevich, Andreas Pittrich, Petja Ivanova, Alissa Maresa Rohrbach, Jakob Kaminski

**Affiliations:** 1 Department of Psychiatry and Neurosciences CCM, 14903 Charité-Universitätsmedizin Berlin, corporate member of Freie Universität Berlin, Humboldt-Universität zu Berlin, and Berlin Institute of Health, Berlin, Germany; 2 Klinik für Psychiatrie und Psychotherapie, Kliniken im Theodor-Wenzel-Werk, Berlin, Germany; 3 Hochschule für angewandte Wissenschaften, Hamburg, Germany; 4 RecoveryCat, Berlin, Germany

**Keywords:** Severe mental illness, schizophrenia, bipolar disorder, patient-reported outcomes, symptom monitoring, user-centered design

## Abstract

**Background:**

The effective treatment of patients with severe psychiatric disorders primarily relies on subjective reporting of symptoms and side-effects. This information is crucial for a clinician's decision regarding medication adjustment. Treatment adjustment usually happens at a low frequency (∼4–8 weeks). In between points of care, patients are left alone with their symptoms and side-effects. This leads to uncertainty regarding the treatment, non-adherence, possible relapse, and rehospitalization.

**Objectives:**

We aim to design a flexible electronic patient-reported outcome (ePRO) system, which allows patients with severe psychiatric disorders to: (a) record their symptoms using an app; (b) share the data with the clinical team at points of care; and (c) utilize the data to support therapy decisions.

**Methods:**

In this article, we describe the development process which included the following steps: (a) formation of a co-design team; (b) stakeholder interviews with patients, practitioners, and digital health experts to access needs, requirements, and barriers; (c) prototype conceptualization and design; (d) user acceptance testing and refinement; and (e) finalization of the system for testing in a pilottrial.

**Results:**

We included input from patients with lived experience of psychiatric disorders, clinical team members, software engineers, and researchers. A prototype system was refined, and iterative changes were made before finalization during a series of operational meetings. The system allows patients to digitally self-report their symptoms and provides longitudinal ePRO symptom data for export into the electronic health record.

**Conclusions:**

Routine ePRO collection has the potential to improve outcomes and hereby also reduce health service costs. We have successfully developed a trial-ready ePRO system for severe psychiatric disorders. The findings were incorporated in the planning of a feasibility pilot trial. Assuming feasibility will be established, the system might be subjected to a certification process evaluation of safety and efficacy including a randomized controlled trial.

## Introduction

Mental illnesses have a high prevalence,^1^ leading to significant impairments on an individual and societal level,^2^ and high treatment costs.^3^ Particularly, severe mental illnesses (SMI), such as bipolar disorder, schizophrenia, major depressive disorder, and anorexia nervosa are characterized by recurring episodes, chronic trajectories, and frequent rehospitalization.^4^

Since treatment response and relapse prediction in mental health are complex, outpatient treatment remains challenging. Outpatient psychiatrists face the task of caring for many patients in routine clinical practice. Consequently, appointments are kept short^5^ and low in frequency leaving patients^5–7^ and clinicians^7^ unsatisfied. From a patient's point of view, summarizing their condition, life events, medication intake and effects, and possible side-effects during a short appointment is challenging. On the other hand, practitioners have to quickly identify interactions and possible correlations between the various factors and draw treatment conclusions (general practitioners use more time for patients with mental health problems than for patients with somatic problems^8^). An app-based companion that allows patients to record their symptoms and medication side-effects in between points of care and that additionally helps to present the data at the next appointment might alleviate these problems by allowing the practitioner to quickly assess symptom trajectories and therefore save time for discussion of the further treatment course.^9^

Formalized reported outcome measures for evaluation treatment response, also referred to as measurement-based care,^10^ are recommended in guidelines and are proven to ameliorate service user activation,^11^ shared decision making,^11,12^ and treatment outcome.^13,14^ Even though the use of patient-reported outcomes (PROs) is increasing, in medicine as well as in psychotherapy^15^ and PROs are applicable in severe mental illness,^16^ PROs are rarely implemented in routine care^17^ due to limited time.^18^

In ambulatory psychiatric care, treatment non-responders as well as side-effects of medication are often not recognized fast enough, which leads to poor patient–physician relationship,^19^ non-adherence to therapy, and ultimately, rehospitalization.^20^ Measurement-based care might help to detect non-responders and relapses earlier and therefore improve outcomes.

While asking patients to regularly complete validated, self-report questionnaires provides a more comparable estimate of symptom change, validated self-reports might not inform about subtle changes in an individual's psychopathology and functioning as well as being burdensome for patients and clinicians.^21^ Moreover, since treatment goals, especially in a recovery-oriented setting, are highly individual, an equally personalized method of tracking goal attainment progress is warranted.^22^ Finally, adherence to digital applications may be higher than to paper-pencil solutions.^23^

Questionnaires filled out during patients’ visits may not provide the necessary temporal resolution for detecting symptom changes or side-effects and are subject to recall bias.^24,25^ Repeated electronic recording of momentary states between points of care (ecological momentary assessment) might provide necessary data for readily detecting and visualizing symptom changes and side-effects of pharmacotherapy—both for patients and for clinicians with high temporal resolution.^26^ This seems to be a suitable way to enable a more accurate recording of psychiatric symptoms with a higher ecological validity and may allow equally precise diagnosis as established rating scales^27^ or might even allow new diagnostic classifications (digital phenotyping^28^).

In this study, we seek to analyze the potential and preconditions for a digital solution that uses the collection of customizable electronic patient-reported outcome (ePROs) to support treatment in psychiatric outpatient clinics (RecoveryCat). We hypothesize that a smartphone-based digital mental health application (DMHI) for recording ePROs has the potential to capture and transfer accurate information on symptom trajectories despite low-frequency and short treatment appointments and that the collected information can be helpful in the process of shared decision making. Specifically, ePROs may allow the detection of subclinical trends in symptoms that act as early relapse predictors. Short-term effects between medication dose adaptation and symptom or medication side-effect changes may become comprehensible. Perspectively, regular symptom tracking may allow differentiation of specific treatment effects and symptom fluctuation due to other causes.^29^

The DHMI for patients and practitioners will help to routinely use ePROs in clinical practice and establish the implementation of the use of an app in clinical practice, putatively increasing sustained use by patients,^30^ eventually for further extensions (e.g. an alert system and a crisis plan connected to the symptoms of the patient).

In this exploratory study, we assess the feasibility and determine key user experience design and app architecture in a multi-stakeholder approach (patients, mental health practitioners, and health insurance providers). Principles of user-centered design were applied both on practitioners’ as well as patients’ side^31,32^ as users were involved in all phases of development (stakeholder interviews, prototype development, and prototype testing). Results of our mixed methods approach and the technical translation of the findings will be presented in a narrative summary. We will discuss possible add-ons and a scope of possible applications.

## Methods

For the development of a flexible, electronically assessed patient-reported outcome tool as described above, we chose the following three phase user-centered design (UCD) research and development strategy.^33^ UCD implies involvement of users at all stages of the development process to ensure needs, preferences, and limitations of the end users are met.^34^

### Setting

The study was conducted in collaboration at the Clinic of Psychiatry and Psychotherapy of Charité Universitätsmedizin Berlin (university clinic) and the Clinic of Psychiatry and Psychotherapy at Kliniken im Theodor-Wenzel-Werk (community psychiatric hospital). The research and development team consisted of psychiatrists, psychologists, and psychotherapists specialized in treatment of patients with severe mental illness (CW, JK, EQ, and TM) as well as a software developer (AP) and user design specialists (PI, AR). The local ethics committee approved the study. After obtaining consent of patients, user design specialists (PI and AR) conducted observational field visits.^32^

### Phase I: stakeholder interviews

The following key stakeholders in the psychiatric care context were identified through user mapping^35^: patients with severe mental illness (n = 11), including patient experts (n = 2, trained peer supporters, “experienced involvement”, for reference, see https://www.ex-in.de), practitioners (n = 11 psychiatrists and psychotherapists in private practice or outpatient clinics), and digital health experts (n = 4, including health insurance companies, digital health consulting companies, and e-mental health startup associates). Inclusion criteria for patients were stable disease and diagnosis of a severe mental illness. Exclusion criteria were age below 18 years and acute crisis (acute suicidal tendencies or necessitating hospitalization).

Semi-structured, qualitative interviews were conducted as UCD procedure to assess unmet needs in the current clinical setting, willingness to use an app, as well as reservations and implementation barriers.^32^ Semi-structured interviews were chosen to ideally generate relevant hypotheses in the field,^35,36^ with a focus on actual experiences during treatment and specific examples. An interview guideline was formulated and used to make the interviews conduction comparable (see supplement file). The interview's length was 30–60 minutes. Relevant information was extracted by the following extraction steps: transcription: First, the interviews were transcribed by the interviewers into a standardized sheet. Core statement summary: Then, relevant statements of each of the interviews were extracted by two team members and documented as core statements (e.g. “regards individualization of questions as necessary”). Coding framework: From the comparison of the core statements of all interviews (AR and PI), recurrent themes were formulated (AR and PI, e.g. “higher adaptability of ePROs preferable”). Coding: Then the transcripts were again analyzed, whether recurrent themes were mentioned. Counting: The number of mentions was counted (AR and PI). The aggregated themes and the number of mentions were handed to the prototype development team (CW, EQ, AP, PI, AR, and JK) to inform the prototype development.

### Phase II: prototype development

The prototype development team defined typical users in the form of personas to give transparency to all team members for whom and which needs the application to be developed for.^37^ The main user insights were then turned into requirements for the app on two levels: the actual features as well as user experience design aspects, such as level of individualization of the questions or user flows. The prototype development team (design in teams^32^) turned the requirements into simple wireframes (application screens without building the actual application technically, first) in order to be reevaluated by interviewed stakeholders.

### Phase III: prototype testing

User acceptance testing of the developed workflow and features was conducted with 11 patients and 11 practitioners at two outpatient clinics using the prototype wireframes. We chose an open testing approach in which we presented wireframes to the users and asked them to think aloud^38,39^ while going through the screens. We observed how users would navigate through the app and understand its functions and usefulness. Iterative changes were made to the ePRO system draft in response to the findings. An iterative user-centered design methodology was repeatedly applied for further refinement of the concept in this early development phase.^40^

## Results

### Stakeholder interviews

In the following section, we will present the central and repeatedly mentioned topics of the stakeholder interviews.

Patients (n = 11) reported feeling stressed and partly overwhelmed before and during a point-of-care situation with their physician (4/11). A lot of information about symptom development, side-effects, and drug intake within a period of weeks needs to be remembered and shared within minutes, in order to provide meaningful data for the practitioner to make a suggestion for future treatment. Our stakeholder interviews revealed that there is a high need for monitoring symptoms and side-effects by patients in order to better understand their own recovery (6/11), have more informed conversations with their practitioners, and detect signs of relapse or crisis early (6/11). Some patients (3/11) already use paper-pencil methods or Excel-sheets for this purpose, but these were described as inconvenient to use during daily routine, and difficult to share with practitioners. Another important finding around symptom monitoring was that questions need to be highly personalized (4/11) and should not only refer to symptoms, but also recovery resources (7/11). Patients reported to have discontinued monitoring because they could not relate to the questions asked or even specific terms used in standardized questionnaires (2/11). A few patients expressed concerns about data privacy (2/11).

Practitioners (n = 11) emphasized the necessity of monitoring applications that are integrated into everyday clinical routine (11/11). Such apps should be easy to use (5/11) and deliver specific and meaningful data (4/11). Some practitioners reported having used digital tools or workarounds with their patients (6/11) for years or even decades to gather data in between points of care, for example asking patients to send daily emails rating their symptom severity (1/11). Our interviewees also stressed the mismatch of traditional paper-pencil rating scales for monitoring symptoms over time (4/11). Concerning the adaptability of the PROs, multiple practitioners mentioned a preference for higher adaptability as opposed to standardization (4/11). Practitioners generally favored DMHIs with integrability into routine outpatient care (5/11). Two practitioners expressed concerns about therapeutic standalone applications, solely used by patients independently of their treatment, because of the intransparency of the parallel therapeutic processes patients engage with (2/11).Our interviews also revealed a clear implementation barrier for our app to be used on a frequent basis by practitioners: A digital tool should not produce extra work in points of care but ideally assist the doctor and therefore be time saving by facilitating information exchange (3/11).

Digital health experts (n = 4) were additionally interviewed to make sure to achieve product-market fit beyond meeting user needs in the future. They were recruited for interviews through the network of the Digital Health Accelerator program of the Berlin Institute of Health, which presupposes relevant past experience in the field of digital health. We surveyed mental health tech founders focusing on SMI. Up to today, we found only two monitoring or journaling tools for patients with SMI: eMoods,^41^ an application for individuals with bipolar disorder and recovery record^42^ for patients with eating disorders. eMoods has around 50,000 active users without marketing activities, proving the need for this target group to follow up their symptoms on a daily basis. Recovery record has about 600,000 users.^42^ Further, regulatory experts stressed the importance of validated ePRO for proving efficacy, next to data safety, and quality control aspects.

### Design of workflow

From the extracted exigencies as described above, the interdisciplinary team designed a workflow with integrability in clinical routine: During the visits in an outpatient clinic, the participating patients choose a common set of questions together with the therapist, which represent individually relevant symptoms from the patient's perspective. In a departure from the usual practice in our psychiatric outpatient clinic of encouraging patients to keep a paper-pencil treatment plan or symptom diary and recording this in the clinical notes, these prompts will be provided for the patient in electronic form. The corresponding questions can have different answer formats: text, continuous scale, interval scale, and multiple choice. A frequency of answering the questions is mutually agreed upon, and different questions can be answered with different frequencies. To give an example: One patient with the diagnosis of paranoid schizophrenia wished to answer a question concerning her sleep duration as a possible relapse indicator every day (answer format: 1–12 hours, discrete scale), while answering questions concerning social withdrawal (answer format: yes/no) and enhanced mistrust once a week (answer format: yes/no). The information gathered was evaluated together with the treating physician. The physician and the patient then mutually decided to reduce the antipsychotic medication.

### System architecture

Because of the sensitivity of the data and the particular and legitimate skepticism of patients and therapists towards digital solutions, data protection is crucial. As key components to address the issue, we chose encryption and decentralized data storage, i.e. insufficiency as a feature: Data only resides on the end user's device and data transmission only happens encrypted and when actively triggered by the user. The app handles two kinds of sensitive data: questions along with answer options and answer schedules on the one hand and the patient's answers on the other hand.

For data transmission, the sender selects the data and the app will generate a QR code that includes a secret key and channel name on our web socket server to allow communication between the web browser and the server. The recipient scans the QR code and both app instances (i.e. the patient and clinician versions) meet on the websocket server. The two parties exchange connection data in order to establish a webRTC connection (Web Real-Time Communication, an open standard for communication from device to device) between the two devices and exit the server. The exchange of the connection data is encrypted with the key found in the QR code. The server cannot read any of the information. The actual user data is then transmitted encrypted through the webRTC connection, again using the secret key found in the QR code. If the webRTC connection cannot be established due to network connectivity issues, the transmission will fall back to transmit the user data through the server in the same way the connection data was transmitted before (as an exception which cannot be induced by the server). In any case, data will be end-to-end-encrypted with the key received through the QR code. The QR code will only be valid for a few minutes. Further use of the data in the electronic health record or hospital information system is planned; respective data privacy regulations apply.

We will develop the core of the software under a permissive open source license (Massachusetts Institute of Technology License). The app will be developed as progressive web app (browser based), in order to reduce access barriers. The ionic framework using angular will be deployed.

### Planning of a case–control pilot trial

After completing the design of the application as described, we plan to test it in a further study. As practitioners described a lack of applications for patients affected by severe mental illness, we are planning a study to investigate the applicability of app-based recording of PROs in patients suffering from bipolar disorder, schizophrenia, schizoaffective disorder, and severe recurrent major depression from clinical practice in two psychiatric institutional outpatient clinics.

**Figure 1:**
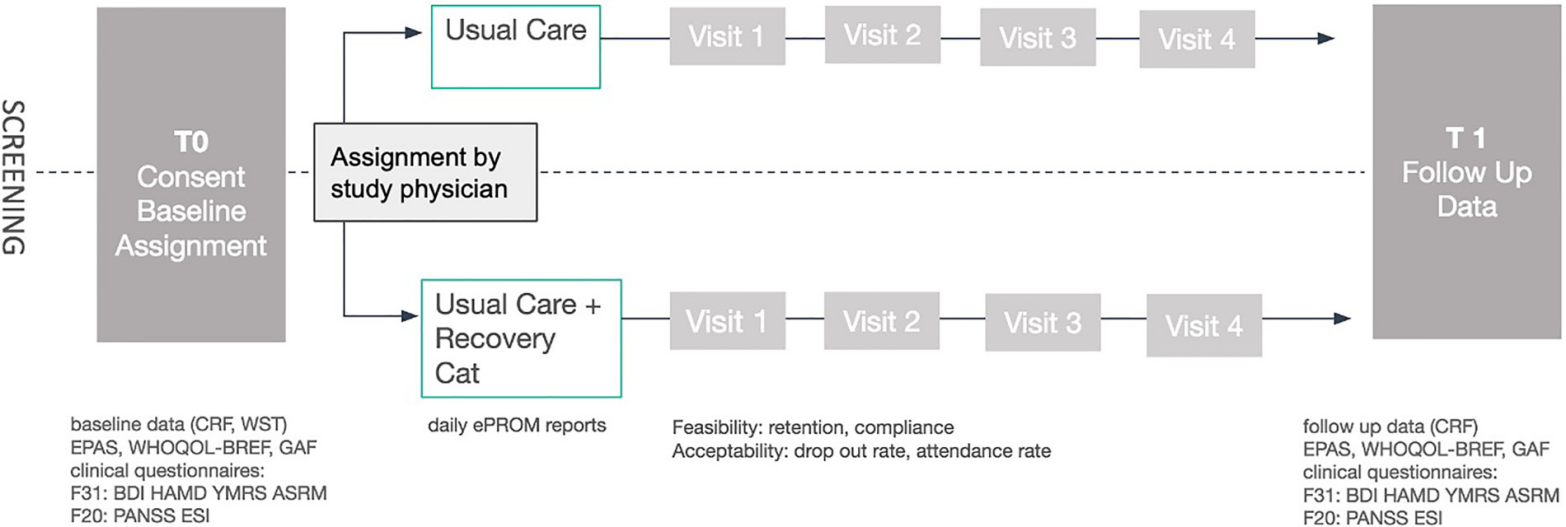
Flow diagram of the planned multicenter case–control study. ARM: Altman Self-Rating Mania Scale; BDI: Beck Depression Inventory; CRF: case report form; ePROM: electronic patient-reported outcome measure; ES: Eppendorfer Schizophrenie Inventar (Eppendorf Schizophrenia Inventory); GAF: Global Assessment of Functioning scale; HAMD: Hamilton Depression Scale; PANSS: Positive and Negative Syndrome Scale; PHQ-9: Patient Health Questionnaire-9; WHO-QoL-Bref: World Health Organization Quality of Life Questionnaire; YMRS: Young Mania Rating Scale.

The pilot study aims first of all to estimate the individual use (adherence, retention rate) and usability (system usability score,^43^ to estimate to what extent certain patient populations can be accompanied with the described procedure. We plan a case–control study design (see Figure 1) for pragmatic reasons, as we expect the design to be the most economic approach to address questions of feasibility and usability. The collected data will also inform the selection of the most suitable primary outcome measures for a future full-scale randomized controlled trial (RCT) and provide data and effect estimates for power calculation to help estimate the sample size for the full-scale RCT with a more refined prototype. We plan to include 60 patients in the intervention group and 60 patients in the control group. Due to the fact that the study is planned as a feasibility study, a power analysis is not applicable. Details on the study including inclusion and exclusion criteria, sample size as well as statistical analysis can be found in the preregistration (DOI 10.17605/OSF.IO/HGKJT; https://osf.io/hgkjt).

Concerning clinical endpoints, we chose indicators of quality of life World Health Organization Quality of Life Questionnaire (WHO-QoL-Bref^44^) and Recovery Quality of Life (ReQoL^45^), the Clinical Global Impression (CGI^46^) for overall symptoms and the Global Assessment of Functioning (GAF) as an indicator of functioning^47^ and the Empowerment-Promoting Assessment Scale (EPAS) for empowerment^48^ in all patient groups. Intelligence was estimated by the Wortschatztest (WST^49^). Diagnosis specific symptoms will be assessed with the Patient Health Questionnaire-9 (PHQ-9),^50^ Beck Depression Inventory (BDI),^51^ Hamilton Depression Scale (HAMD)^52^ for depression, Positive and Negative Syndrome Scale (PANSS)^53^ and Eppendorfer Schizophrenie Inventar (ESI)^54^ for psychosis, and Young Mania Rating Scale (YMRS)^55^ and Altman Self-Rating Mania Scale (ASRM^56^) for manic episodes. Self-rating scales and clinical rating scales were chosen to explore correlations of our ePRO items with self-assessment and clinical assessment. The approval by the Charité's ethics board was obtained for the conduct of the study.

## Discussion

### Principal results

The stakeholder interviews confirmed our main hypothesis on the therapeutic potential of ePROs through a smartphone-based application in outpatient care for the treatment of patients with severe mental disorders. We obtained highly relevant information, mostly in line with previous findings concerning usability,^39^ data security,^30,57^ flexibility, and need for treatment implementation^39,58,59^ which was used to define a workflow in routine care, and design the user interfaces and the app architecture. Our concept of integration of ePRO data in outpatient visits in which practitioner and patient discuss ePRO results has been shown to be the probably most effective form of feedback: A scoping review comparing ePRO interventions with varying degrees of clinician feedback found the largest proportion of studies with statistically significant outcomes amongst studies that provided “formalized mechanisms” to guide ePRO feedback discussions.^60^

The remarkable success of apps like eMoods,^41^ created with a minimum budget for bipolar disorder, already shows the demand of patients to monitor their symptoms and better understand their disease, even though the application does not interact with psychiatric outpatient treatment and covers only one symptom group. The integration of such app-based approaches into psychiatric or psychotherapeutic care therefore seems promising.

### User involvement

Continuous patient involvement, as recommended by the National Institute of Mental Health^61^ in prototype evaluation, was crucial to affirm and refine our conception. While interviews with practitioners mostly corroborated our concept, patient interviews led us to unexpected needs (monitoring to better understand their condition and monitoring achievements and successful recovery strategies).

To make sure the application really meets the needs of the future user group, we will involve a patient advisory board to conduct co-creation sessions and include an individual with previous psychosis experience into our research group to further build a user-centered organizational culture.^32^ Ideally, user involvement should not only include individuals who are already using mental health services^33^ but also people who are currently not using mental health service for different reasons, e.g. related to limited resources or rejection of the available healthcare facilities. Since we recruited all participants from mental health service users, some potential user needs might not be sufficiently addressed, limiting the generalizability of our finding.

We hypothesize that intensive user involvement will help to increase retention rates in future development phases and will become standard procedure in future mental health application development, content creation, and research, since insufficient adaptation of DMHIs to user needs is a barrier to their deployment in real-world healthcare settings.^62,63^

### Limitations

Since our ePRO system is currently a prototype, its usability and the feasibility of the use in routine care as well as its therapeutic effects will have to be further studied. Adherence to the system on the patient side, especially in case of episodes of relapse of severe mental illness, might prove challenging to achieve, even though access to smartphone technology by individuals suffering from mental illness is improving.^30^ On the practitioners’ side, we will be equally challenged to refine our system to be easily and rapidly integrated in routine care with the limited time frames in private practice and outpatient clinics.^58^ Implementing the ePRO system output into different systems of electronic health records is warranted. Therefore, application programming interfaces (API) to electronic health records (EHR) are necessary. We plan to implement Fast Healthcare Interoperability Resources (FHIR) and Systematized Nomenclature of Medicine Clinical Terms (SNOMED-CT) compliant interoperable standards in order to facilitate implementation.

The application should not only be suitable for the technology affine practitioners and patients. Our planned case–control will address these questions and be a testing ground for possible further adaptations.

### Potential benefits for pharmacotherapy

If the proposed ePRO solution proves feasible in the planned case–control study, future patients and users could benefit from more dense data to inform treatment, especially during pharmacotherapy.

Specifically, it would allow for faster detection of response and non-response to drugs and consequently result in a faster change to a more effective therapy with shorter disease phases. Also, more precisely adjusted medication (use of minimum effective dose) could lead to fewer adverse drug reactions. Similarly, adverse drug effects could be detected earlier, reducing exposition. Medication might also be spared entirely through earlier detection of relapses, allowing faster therapeutic response to prevent inpatient admission and the need for intensified pharmacotherapy. Finally, the ePRO solution may provide a better understanding of individual non-adherence, reducing delays in therapy adaptation due to unreported non-adherence.

### Potential benefits and disadvantages of flexible ePROs

User research led to the design of a DHMI used by patients and practitioners in a collaboration, which might be beneficial for therapeutic alliance.^64^ Predefined psychometric rating scales inevitably lead to evaluating variables irrelevant to patients’ specific circumstances.^22^ The joint formulation of relevant outcome measures may stimulate a dialogue between practitioner and patient, which will presumably lead to better understanding of patient preferences.^58^ Another possible approach to adjusting ePRO to individual patients is the continuous reduction of items through algorithms during their use.^65^

Strand et al.^65^ describe the support of individual goals as one common, underlying theme of e-recovery DHMIs for SMI. The shift from rigid outcome measures to individual need-centered care is a core concept of successful psychiatric treatment models for severe mental disorders like Finnish “Open Dialogue” approach^66^ and the German “Weddinger Modell”.^67^ Our focus on flexibility and adaptability has conceptual overlap with the goal attainment scale^22^ and the Psychological Outcomes Profiles (PSYCHLOPS).^68^

In studies along the lines of Open Dialogue,^69^ the use of the patient's own words for the patient's symptoms has shown to contribute significantly to illness insight and understanding, and is thus essential for therapeutic alliance and ultimately recovery. Similar approaches are being developed in the field of psychotherapy, as patients are asked to define relevant outcome parameters in their own words.^70^

A flexible ePRO monitoring should therefore be readily available within minutes and more centered around patients’ goals and needs and less towards the practitioner's diagnostic ambitions. Herein, our concept differs from preexisting solutions.^71–75^ We hypothesize that radical adaptation to user's needs will enhance user retention rates, since attrition rates are high, if an ePRO system does not address relevant symptoms or users perceive a mismatch between usefulness and needed time resources.^76^ Our finding that practitioners also prefer highly adaptable MBC solutions is in line with previous findings.^59^ On the other hand, individualized ePROs might not offer the reliability, objectivity, and validity of standardized questionnaires. Individualized ePROs cannot simply be compared between individuals, reducing the scientific value of gathered outcome data, but might still show good correlation with established outcome measures.^68^

### Potential for further research

RCTs with standardized rating scales are still regarded as cornerstone of outcome research for drugs as well as for psychotherapy outcome research, however, app-based patient-reported outcomes are gaining more and more attention from assessment of quality of life (relevant to value based care) to more fine grained symptom assessment.^77^ Ecological momentary assessment during ongoing treatment could be seen as repetitive measures of outcomes and serve as a robust data basis for n-of-1 trials. n-Of-1 experiments are a necessary extension to RCTs in the development of individualized medicine,^78^ especially in psychiatry, since the possibilities of prediction of response to antidepressants,^79^ antipsychotics,^80^ and psychotherapy^81^ are still very limited and the occurrence of side-effects is variable.^82^ Our ePRO system might transform everyday clinical care into solid n-of-1 trials as a necessary extension to randomized controlled studies and serve as a basis for individualized medicine.

## Outlook

In this article, we focused on the effects in psychiatric outpatient care. Since feedback mechanisms have proven efficient in psychotherapy, individual or group psychotherapy would be another very promising area of application of adaptable digital ePRO systems.^14,81,83^

A future integrated mental health app could additionally include crisis alerts, a crisis plan,^84^ a medication scheme, and provide information on psychiatric illness and treatment options. Digital tools in general allow for a widespread use of expert knowledge also in underserved regions.^85^ This could enable patients to better assess their own state of health, and also to better monitor the progress and success of the medication and interventions undertaken.

In the future, specialized ePROs might be used and regularly responded to by patients undergoing different forms of interventions e.g. by primary care physicians or other specialists and thus enhance the tool with the expertise of other physicians. The digital solution should also aim to integrate caregivers on a permanent basis to provide a more precise perspective on the reality of patients’ lives in their social environment outside the clinic. Since patients would carry relevant information with them, while being in treatment in an inpatient unit, day clinic, specialized outpatient clinic, or at their general practitioner, digital tools possibly can reduce the negative effects of fragmentation of the mental healthcare systems by enhancing patients’ agency over their treatment trajectories.

Ultimately, digital tools might stimulate research by providing meaningful and valuable real-world data to researchers with limited resources for funding large RCTs for evaluating mental health interventions.

## Conclusions

Our user-centered design study led to the development of a highly adaptable ePRO system for outpatient treatment of patients with severe psychiatric disorders. The highly flexible ePRO collection application has the potential to improve how treatment is delivered in psychiatry. The feasibility of the application will be evaluated in a planned trial.

## Supplemental Material

sj-docx-1-dhj-10.1177_20552076231191009 - Supplemental material for A digital patient-reported outcome (electronic patient-reported outcome) system for patients with severe psychiatric disorders: User-centered development study and study protocol of a multicenter-controlled trial Click here for additional data file. Supplemental material, sj-docx-1-dhj-10.1177_20552076231191009 for A digital patient-reported outcome (electronic patient-reported outcome) system for patients with severe psychiatric disorders: User-centered development study and study protocol of a multicenter-controlled trial by Caspar Wiegmann, Esther Quinlivan, Twyla Michnevich, Andreas Pittrich, Petja Ivanova, Alissa Maresa Rohrbach and Jakob Kaminski in DIGITAL HEALTH
