# Supplementary material for: A digital patient-reported outcome (electronic patient-reported outcome) system for patients with severe psychiatric disorders: User-centered development study and study protocol of a multicenter-controlled trial
Source: Digit Health. 2023 Oct 25;9:20552076231191009. doi: 10.1177/20552076231191009 (PMC10605665; doi:10.1177/20552076231191009)
Supplement: sj-docx-1-dhj-10.1177_20552076231191009 - Supplemental material for A digital patient-reported outcome (electronic patient-reported outcome) system for patients with severe psychiatric disorders: User-centered development study and study protocol of a multicenter-controlled trial [file sj-docx-1-dhj-10.1177_20552076231191009.docx]

**Stakeholder Interview Guideline**

[**A) Patients**](#_pa5i9erdq334) **1**

[**B) Health care professionals**](#_vlkrjaldumif) **1**

[**C) Digital health experts and health plan providers**](#_o0wcqm577n9x) **2**

### A) Patients:

0. Intro

- Introduce the research project and the research team
- Explain user centered design method
- Explain goal of the interview: to understand how patient feels in psychiatric outpatient care, especially between appointments and with a focus on symptom trajectories
- Explain importance of critical feedback
- Explain confidentiality and anonymization of the interview

1. Open questions

- Can you tell me about yourself and therapy context?
- How often do you see your doctor and how do these appointments work?
- What is important for you in your treatment?
- How satisfied are you with your outpatient treatment? How well are your needs met in the appointments?
  - Why exactly is it important? What does it mean to you?

2. In-depth questions/ Evaluation of needs & pain points

- What could go better in regards to your outpatient treatment?
- Can you explain a concrete example?
  - Why was this stressful to you?
  - What was the result?
  - Did you try to find solutions for this and if yes, how?

3. Wishes for a solution

- What would help to better solve the problem mentioned?
- What could this look like?

4. Ending & Open thoughts

- Would you like to mention anything else we didn't cover regarding the challenges in treatment or potential solution?
- Explain what happens to the interview results
- Exchange again contact details in case of further feedback or questions

###

###

### B) Health care professionals

0. Intro

- Intro about the research project and the team
- Explain user centered design method
- Explain goal of the interview: understand how patient feels in psychiatric outpatient care, especially between appointments and with a focus on symptom trajectories
- Explain how important critical feedback is
- Explain confidentiality and anonymization of the interview

1. Open questions

- Can you tell me about yourself and your daily work as a HCP?
- How often do you see your patients and how do these appointments work?
- What is important for you regarding the treatment you provide?
- How satisfied are you with the outpatient treatment you provide?
  - Why exactly is it important? What does it mean to you and your patients?

2. In-depth questions/ Evaluation of needs & pain points

- What could go better in regards to the treatment you provide?
- Can you explain a concrete example?
  - Why was this stressful to you?
  - What was the result?
  - Did you try to solve it and if yes, how?

3. Wishes for a solution

- What would help to better solve the problem mentioned?
- What could this look like?

4. Ending & Open thoughts

- Would you like to mention anything else we didn't cover regarding the challenges in treatment or potential solution?
- Explain what happens to the interview results
- Exchange again contact details in case of further feedback or questions

###

###

###

###

###

### C) Digital health experts, including health plan providers

0. Intro

- Intro about the research project and the team
- Explain user centered design method
- Explain goal of the interview: understand how patient feels in psychiatric outpatient care, especially between appointments and with a focus on symptom trajectories
- Explain how important critical feedback is
- Explain confidentiality and anonymization of the interview

1. Open questions

- Can you tell me about yourself and your work in the field of digital health/ healthcare?
- How are you/is your company involved in psychiatric outpatient care?
- How do you see the space (psychiatric outpatient care, digital solutions) right now?

2. In-depth questions/ Evaluation of needs & pain points

- What would you wish to change about the current situation in psychiatric outpatient care?
- Why (why is it a problem)?

3. Wishes for a solution

- What would help to better solve the problem mentioned?
- What could this look like?

4. Ending & Open thoughts

- Would you like to mention anything else we didn't cover?
- Explain what happens to the interview results
- Exchange again contact details in case of further feedback or questions
